# Lipoic Acid as a Possible Pharmacological Source of Hydrogen Sulfide/Sulfane Sulfur

**DOI:** 10.3390/molecules22030388

**Published:** 2017-03-02

**Authors:** Anna Bilska-Wilkosz, Małgorzata Iciek, Danuta Kowalczyk-Pachel, Magdalena Górny, Maria Sokołowska-Jeżewicz, Lidia Włodek

**Affiliations:** Chair of Medical Biochemistry, Jagiellonian University Collegium Medicum, 7 Kopernika Street, 31-034 Kraków, Poland; miciek@cm-uj.krakow.pl (M.I.); dkowalczyk@cm-uj.krakow.pl (D.K.-P.); mbgorny@cyf-kr.edu.pl (M.G.); marsokol@wp.pl (M.S.-J.); mbwlodek@cyf-kr.edu.pl (L.W.)

**Keywords:** lipoic acid, dihydrolipoic acid, sulfane sulfur, hydrogen sulfide

## Abstract

The aim of the present study was to verify whether lipoic acid (LA) itself is a source of H_2_S and sulfane sulfur. It was investigated in vitro non-enzymatically and enzymatically (in the presence of rat tissue homogenate). The results indicate that both H_2_S and sulfane sulfur are formed from LA non-enzymatically in the presence of environmental light. These results suggest that H_2_S is the first product of non-enzymatic light-dependent decomposition of LA that is, probably, next oxidized to sulfane sulfur-containing compound(s). The study performed in the presence of rat liver and kidney homogenate revealed an increase of H_2_S level in samples containing LA and its reduced form dihydrolipoic acid (DHLA). It was accompanied by a decrease in sulfane sulfur level. It seems that, in these conditions, DHLA acts as a reducing agent that releases H_2_S from an endogenous pool of sulfane sulfur compounds present in tissues. Simultaneously, it means that exogenous LA cannot be a direct donor of H_2_S/sulfane sulfur in animal tissues. The present study is an initial approach to the question whether LA itself is a donor of H_2_S/sulfane sulfur.

## 1. Introduction

Lipoic acid (LA, 1,2-dithiolane-3-pentanoic acid) was first identified by Reed et al. [1]. In mammals, it is synthesized in very small quantities in the liver and other tissues and is used as a cofactor of mitochondrial complexes catalyzing oxidative decarboxylation of alpha keto acids and glycine cleavage system (GCS) also localized in mitochondria. In these enzymatic complexes, LA is linked by an amide bound to the ε-amino group of a lysine residue of the protein.

Apart from endogenous synthesis, LA is also absorbed from exogenous sources and can be reduced enzymatically in the body to dihydrolipoic acid (DHLA, 6,8-dimercaptooctanoic acid). Typical dietary sources of LA include meat offal and vegetables. Moreover, LA has now become a common ingredient of different multivitamin formulas, dietary supplements and even pet food [2]. This compound has also gained the attention of cosmetologists and dermatologists since available data indicate a positive effect of LA-containing preparations (creams, ointments, etc.) on the skin [3]. In the last several years, the interest of researchers has focused mainly on the therapeutic properties of exogenously supplemented LA. In many countries, LA is approved as a drug against diabetic neuropathy and is available by prescription [4]. Our studies have proven a beneficial effect of LA in Parkinson’s disease, kidney disease, nitroglycerin tolerance, cyanide and cyanate intoxication [5,6,7,8,9]. Many authors have suggested that therapeutic effects of LA supplementation can be attributed to the potent antioxidant properties of LA and DHLA [10,11,12]. However, other authors question the efficacy of the supplemented LA as a physiological antioxidant because the action of LA/DHLA as direct scavengers of reactive oxygen/nitrogen species (ROS/RNS) was confirmed mostly in in vitro experimental studies [13]. Thus, although therapeutic efficacy of LA, especially in the treatment of diabetes and diabetic neuropathy, is beyond doubt, the cellular and biochemical mechanisms that mediate the pharmacological effects of exogenous LA are yet to be uncovered.

Almost 10 years ago, we hypothesized for the first time that therapeutic effects of LA are due to their involvement in sulfane sulfur metabolism [14]. Iciek et al. indicated that LA supplementation led to augmentation of the sulfane sulfur level in erythrocytes of end-stage renal failure patients treated with peritoneal dialysis [15]. The study in a mouse peritonitis model indicated that LA increased sulfane sulfur level in the peritoneal exudates [16]. The animal studies using glibenclamide, an adenine triphosphate (ATP)-sensitive potassium channel (K_ATP_) blocker, clearly demonstrated that the anti-inflammatory [17] and cardioprotective [18,19] properties of LA were associated with the action of H_2_S.

Therefore, the question arises whether the therapeutic effects observed in vivo after LA administration can be associated with its influence on endogenous H_2_S/sulfane sulfur production or whether exogenous LA itself can act as a direct donor of H_2_S/sulfane sulfur.

The process of H_2_S release from sulfides (RSH) and disulfides (RSSR) has been studied for many years. It is known that the reaction of the hydrated electron with RSH leads to breakdown of C–S bond and formation of H_2_S and a carbon-centered radical (R^•^) whilst disulfides (RSSR) react with the hydrated electron forming sulfur-centered radical species [20,21]. LA is a very unique molecule due to its characteristic five-membered dithiolane ring which, in contrast to linear disulfide in cystine, is unstable under ultraviolet irradiation [22]. Therefore, several in vitro chemical and photochemical studies of LA have focused mainly on the products of cleavage of its disulfide bond, producing reactive thiyl radicals [23,24,25]. In contrast, processes of breakdown of the C–S bond in the LA molecule have been less studied.

The aim of the present study was to answer the question of whether H_2_S/sulfane sulfur can be released from an LA molecule. Non-enzymatic and enzymatic H_2_S and sulfur formation from LA was investigated in vitro in the absence of homogenate and in the presence of rat liver or kidney homogenate, respectively.

In in vitro conditions, in the absence of enzymes, only the oxidized form, i.e., LA is stable. The reduced form, i.e., DHLA is, under these conditions, very quickly oxidized to LA. For this reason, the oxidized form, i.e., LA, is administered as a drug. It is assumed that, after administration, LA is reduced in the body to DHLA by three enzymes: dihydrolipoate dehydrogenase, glutathione reductase or thioredoxin reductase [26]. Thus, both forms of this compound—LA and DHLA—are present in vivo while, in vitro, only LA is stable. This is the reason why in the studies conducted in the presence of rat tissue homogenate, both forms LA and DHLA were used, whereas, in the absence of rat tissue homogenate, we applied only LA.

## 2. Materials and Methods

### 2.1. Chemicals

The drug lipoic acid (Thiogamma^®^ 600) was obtained from Wörwag Pharma Company (Böblingen, Germany). Dihydrolipoic acid, sodium sulfide (Na_2_S), *p*-phenylenediamine, potasium cyanide (KCN), 5,5’-dithio-bis-2-nitrobenzoic acid (DTNB) and zinc acetate were provided by Sigma Chemical Co. (St. Louis, MO, USA). Formaldehyde, ferric chloride (FeCl_3_) and other chemicals were purchased from the Polish Chemical Reagent Company (P. O. Ch, Gliwice, Poland).

### 2.2. Animals and Homogenate Preparation

One male Wistar rat was used to obtain liver and kidney homogenate. An animal (weighing approximately 200 g) was kept under standard laboratory conditions and was fed a standard feed. It was sacrificed by decapitation, the liver and the kidneys were isolated, placed in liquid nitrogen and stored at −76 °C until the experiment. The procedure was approved by the Animal Research Ethics Committee in Krakow, Poland (number 153/2012).

The frozen liver or kidneys were weighed and immediately homogenized (1 g of the tissue in 4 mL of 0.1 M phosphate buffer, pH 7.4) using an IKA-ULTRA TURRAX T8 homogenizer (IKA Poland Sp. z o.o company, Warsaw, Poland) (6000 rpm for 1 min). Homogenates were kept in ice and used immediately for experiments.

### 2.3. Hydrogen Sulfide and Sulfane Sulfur Formation from Lipoic Acid

Reactive sulfur species (H_2_S and sulfane sulfur) formation from LA was investigated in vitro non-enzymatically (without homogenate). Samples of 10 mM LA in 50 mM phosphate buffer, pH 5.0 and 8.0 were prepared and were left at room temperature for 3 or 7 days protected or unprotected from environmental light. Independently, on the last day of experiment, fresh samples of 10 mM LA in phosphate buffer, pH 5.0 and 8.0 were prepared. Then, both LA samples (“old” and “fresh”) were used for H_2_S and sulfane sulfur determination. In some experiments, ethylenediaminetetraacetic acid (EDTA) was included in the buffer.

Possibilities of H_2_S/sulfane sulfur formation from LA/DHLA were also investigated in vitro enzymatically in the presence of fresh rat liver or kidney homogenate (protected from light).

### 2.4. Methods

#### 2.4.1. Determination of H_2_S Level

H_2_S level was determined using a modification of the method described by Shen et al. [27]. Briefly, 125 μL of LA samples and 125 μL of water (or 125 μL of LA sample and 125 μL of rat liver homogenate) were mixed with 125 μL of 1% zinc acetate and 125 μL of 50 mM borate buffer, pH 9.0 and the mixture was incubated at 37 °C for 10 min. Next, 0.4 mL of 12.5 mM *p*-phenylenediamine and 0.1 mL of 40 mM FeCl_3_ in 6 M HCl were added. After a 10 min incubation at room temperature, the samples were centrifuged at 10,000× *g* for 5 min, and fluorescence was measured at wavelength λ= 600 nm for excitation and λ = 623 nm for emission H_2_S concentrations were read from a calibration curve prepared from 10 μM Na_2_S and were expressed in nmoles of S^2−^ per 1 mL.

#### 2.4.2. Determination of Sulfane Sulfur

The level of the compounds containing sulfane sulfur was determined by the method of Wood based on cold cyanolysis and colorimetric detection [28].

Initially, 200 μL of LA sample and 620 μL of distilled water (or 200 μL of LA sample, 200 μL of homogenate and 420 μL of distilled water) were mixed with 80 μL of 1 M NH_3_, and 100 μL of 0.5 M KCN. Then, the mixtures were mixed thoroughly and incubated at room temperature for 45 min. Next, 20 μL of 38% formaldehyde solution and 200 μL of the Goldstein reagent containing Fe^3+^ cation were added. The precipitate formed in this reaction was centrifuged at 10,000× *g* for 10 min. The supernatant was carefully collected and its absorbance was measured at a wavelength λ = 460 nm. The whole pool of sulfane sulfur was evaluated from a standard curve prepared for 1 mM KSCN and was expressed in nmoles of KSCN per 1 mL.

#### 2.4.3. Determination of H_2_S by Diffusion Assay

H_2_S liberated from LA was determined by the modified diffusion assay described by Toohey [29]. Special diffusion chambers contain a space in the middle separated from the sample. The chambers are tightly closed and gas liberated from samples can diffuse and react with the reagents inside the cell. The inner cell of the chamber contained 0.9 mL of 0.3 M Na_2_PO_4_ and 0.1 mL of 6 mM DTNB, while, to the outer cell of the chamber, 1 mL of LA sample was added. The diffusion chambers were immediately closed and placed on a shaker. The diffusion was carried out for 90 min, and then the absorbance of the solution located in the inner cell was measured spectrophotometrically against the blank sample containing only DTNB and buffer. H_2_S liberated from the outer part of the chamber reacted with DTNB in the inner part to form 2-nitro-5-thiobenzoic acid characterized by intensive yellow color, which shows maximum absorbance at 412 nm. The H_2_S level was evaluated from a standard curve prepared for 10 μM Na_2_S and was expressed in nmoles per 1 mL.

### 2.5. Statistical Analysis

All experiments were performed in triplicate or duplicate. Results are presented as the means ± SD (standard deviation). A statistical analysis of experimental data was performed using a one-way analysis of variance ANOVA. The differences were considered statistically significant when *p* < 0.05. The statistical analysis was done using STATISTICA 12.0 Software (Statsoft Inc., Tulsa, OK, USA).

## 3. Results

### 3.1. Hydrogen Sulfide Formation from Lipoic Acid

**Figure 1:**
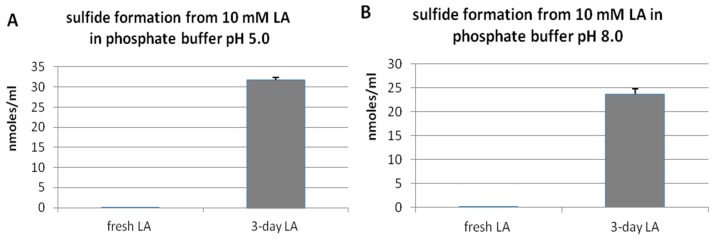
Level of hydrogen sulfide formed in vitro from fresh lipoic acid (LA) or from LA left for three days unprotected from light at pH 5.0 (**A**) or 8.0 (**B**). The results are presented as the means ± SD (standard deviation).

Non-enzymatic H_2_S formation from LA was investigated in vitro in a buffer environment. LA samples were prepared in phosphate buffer at two pHs: 5.0 and 8.0. Figure 1 demonstrates that freshly prepared LA solution, if protected from light, contained only trace amounts of H_2_S independently of pH conditions. However, when LA samples were left for three days at room temperature and unprotected from light, the amount of H_2_S strongly increased. These results suggest that, in the presence of light, LA can be decomposed non-enzymatically with H_2_S release.

### 3.2. Sulfane Sulfur Formation from Lipoic Acid

**Figure 2:**
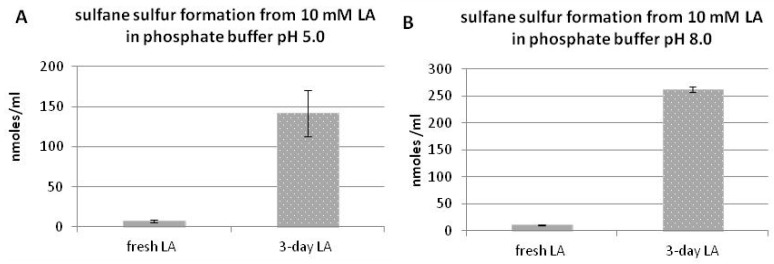
Level of sulfane sulfur formed in vitro from fresh LA or from LA left for three days unprotected from light at pH 5.0 (**A**) or 8.0 (**B**). The results are presented as the means ± SD.

Figure 2 presents non-enzymatic sulfane sulfur formation from LA under in vitro conditions. The fresh LA contained trace amounts of sulfane sulfur; however, after three days, significant amounts of sulfane sulfur were detected in the samples non-protected from light. A slightly higher level of sulfane sulfur was estimated at pH 8.0 (Figure 2A,B). It suggests that sulfane sulfur is formed from LA non-enzymatically in the presence of light.

### 3.3. pH- and Time-Dependent H_2_S Formation from Lipoic Acid Detected by Diffusion Assay

**Figure 3:**
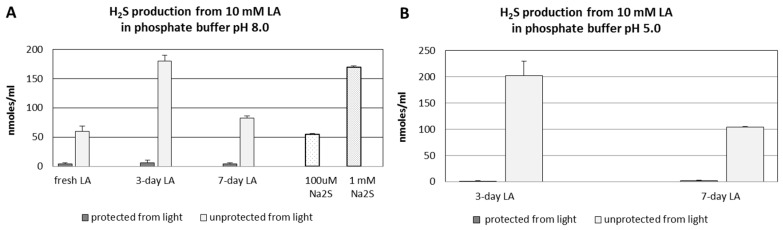
Level of H_2_S formed in vitro non-enzymatically from LA protected or non-protected from light detected by diffusion assay. In addition, 10 mM solution of LA in 50 mM phosphate buffer was used fresh as well as after three or seven days left in the presence or absence of environmental light. On the day of the experiment, the LA samples were pipetted into diffusion chambers and the level of H_2_S was detected after 90 min of diffusion. (**A**) the level of H_2_S formed at pH 8.0 from fresh LA, three-days LA and seven-days LA as well as from 100 µM and 1 mM Na_2_S as the controls; and (**B**) the level of H_2_S formed at pH 5.0 from three-days LA and seven-days LA. The results are presented as the means ± SD from samples run in triplicate.

Figure 3A,B present the results of non-enzymatic H_2_S formation from LA detected by the diffusion assay. Figure 3A represents H_2_S formation from LA at pH 8.0 dependent on time and light protection in comparison to the standard solutions of Na_2_S. When LA was protected from light all the time (during the diffusion assay also), only trace amounts of H_2_S were detected independently of the time (the same level for fresh, three-days and seven-days LA solution). However, when the samples of the same LA solution were not protected from light, significant levels of H_2_S were determined after three and seven days at pH 8.0 (Figure 3A) and 5.0 (Figure 3B). Moreover, a noticeable amount of H_2_S was detected even from fresh LA, when 90-min diffusion assay was non-protected from light. (Figure 3A). The amounts of H_2_S liberated from three-days LA were the highest both at pH 8.0 and 5.0 (Figure 3A,B). Interestingly, the results of this experiment demonstrated that H_2_S liberated from seven-days LA left exposed to environmental light was nearly twice the amount lower than three-days LA samples at both pH values.

### 3.4. Time-Dependent Sulfane Sulfur Formation from Lipoic Acid in the Presence or Absence of EDTA

**Figure 4:**
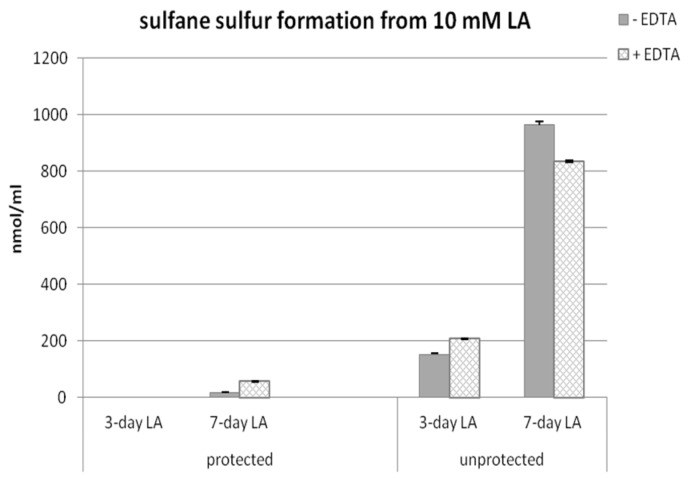
Level of sulfane sulfur formed in vitro non-enzymatically from fresh LA protected from light, and LA left for three or seven days unprotected from light in the presence or absence of ethylenediaminetetraacetic acid (EDTA).

Figure 4 presents results of sulfane sulfur determination in the samples of LA left for three or seven days protected or non-protected from light in the presence or absence of EDTA.

When samples were protected from light independently of the presence of EDTA, no or only a trace amount of sulfane sulfur was detected after three or seven days, respectively. A significant amount of sulfane sulfur was detected only in samples non-protected from light, especially after seven days. After three days, the level of sulfane sulfur in the samples left unprotected from light was much smaller than after seven days but visibly higher than in samples protected from light. EDTA in three-day LA samples raised subtly the amount of sulfane sulfur, but, in seven-day LA samples, the level of sulfane sulfur was a slightly decreased in EDTA samples (Figure 4). The presence of EDTA had no clear effect on the level of sulfane sulfur.

These results together with the results presented in Figure 3 suggest that H_2_S is the first product of non-enzymatic light-dependent decomposition of LA, which then is probably oxidized to sulfane sulfur-containing compounds.

### 3.5. H_2_S and Sulfane Sulfur Formation from Lipoic Acid or Dihydrolipoic Acid in the Presence of Rat Liver or Kidney Homogenate

**Figure 5:**
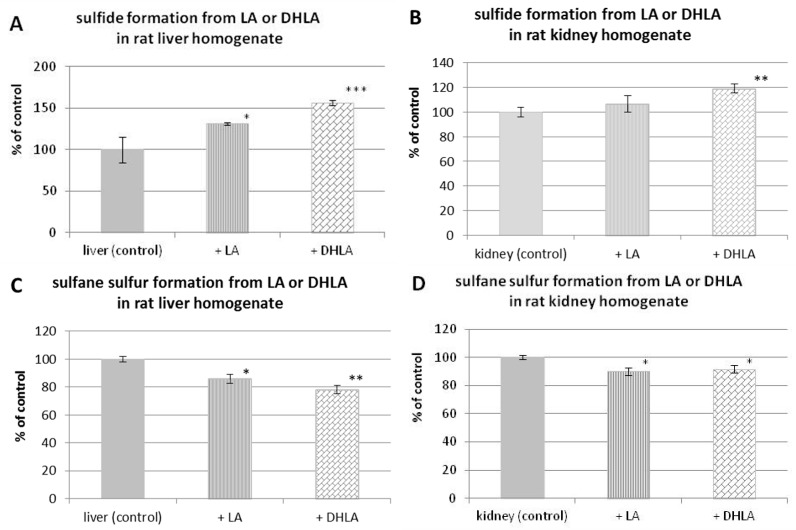
Level of hydrogen sulfide (**A**,**B**) and sulfane sulfur (**C**,**D**) formed in vitro enzymatically in the presence of rat liver or kidney homogenate from fresh LA or dihydrolipoic acid (DHLA) protected from light compared to endogenous hydrogen sulfide and sulfane sulfur level in homogenates (control). The results are presented as the means ± SD. Statistical significance compared to the control (* *p* < 0.05; ** *p* < 0.01; *** *p* <0.001).

The possibilities of enzymatic H_2_S formation from LA in the absence of light are presented in Figure 5. To the rat liver or kidney homogenate, freshly prepared LA or DHLA solutions were added and samples were incubated at room temperature for 30 min completely protected from light. Then, H_2_S level and sulfane sulfur level were determined and compared to the control homogenate. Figure 5A demonstrates that the amount of H_2_S is significantly higher in the samples of rat liver homogenate containing LA or DHLA than in control homogenate. A greater amount of H_2_S was formed from the same molar concentration of DHLA than LA in the sample. Similar results were obtained in the experiment performed in the presence of kidney homogenate; however, in this case the amounts of H_2_S were lower than in the case of liver homogenate and only DHLA caused a significant increase in H_2_S level (Figure 5B).

The results obtained from sulfane sulfur determinations in samples containing rat homogenate and LA or DHLA in the absence of light are presented in Figure 5C,D. Interestingly, the levels of sulfane sulfur compounds in control liver or kidney homogenates were higher than in samples containing the same amounts of homogenate and LA or DHLA (Figure 5C,D). Moreover, the greatest decrease in sulfane sulfur level was found after DHLA treatment, where the highest increase in H_2_S level was also observed. It means that the increase in H_2_S level observed after addition of DHLA/LA is connected with its release from endogenous sulfane sulfur compounds under the influence of DHLA.

## 4. Discussion

It is known that short-wavelength UV (UVC: 200 nm–280 nm) irradiation facilitates breakage of C–S bond in thiol compounds and H_2_S release [30]. LA that is a very unique dithiol molecule [31] has not been studied in this context for a long time, until the study of Bucher et al., who reported, for the first time, that C–S bonds in LA molecules can be broken forming carbon-centered radicals and H_2_S under the influence of 266 nm irradiation [32]. The aim of our study was to verify if this process can be initiated by natural sunlight (environmental light) and the obtained results confirmed this possibility. It is commonly known that ground level sunlight is composed of 44% visible light, 3% ultraviolet and the remainder is infrared radiation. The UVC is completely absorbed by the ozone layer and atmosphere. Therefore, 95% or more of UV radiation that reaches the Earth’s surface is composed of the longer wavelengths: UVA (315 nm–400 nm) with the small remainder of UVB (280 nm–315 nm). In general, it can be concluded that the UV radiation that reaches the Earth’s surface does not contain the UVC component. Moreover, the ordinary window glass blocks over 90% of the radiation below 300 nm.

LA is a very unique molecule due to its characteristic five-membered dithiolane ring which, in contrast to linear disulfide in cystine, is unstable under ultraviolet irradiation [22]. Therefore, several in vitro chemical and photochemical studies of LA have focused mainly on the products of cleavage of its disulfide bond, giving reactive thiyl radicals [23,24,25].

Thus, in this study, H_2_S/sulfane sulfur release from LA molecules under the influence of visible light was observed for the first time. We assume that in our experimental conditions the C–S bonds are broken in the LA molecule to yield H_2_S. The present results indicate that not only H_2_S but also sulfane sulfur is formed from LA non-enzymatically under in vitro conditions in the presence of visible light (Figure 2).

H_2_S is released from LA molecules in a time- and light-dependent manner. Only trace amounts of H_2_S were detected both in freshly prepared LA solution, and in LA samples, which were left three or seven days protected from light (Figure 1, Figure 3). In this context, the results of non-enzymatic H_2_S formation from LA detected by diffusion assay are especially interesting as they indicated that H_2_S production in seven-day LA samples was much lower than in three-day LA samples (Figure 3). Simultaneously, sulfane sulfur production in seven-days LA samples was much higher compared to sulfane sulfur level detected in three-days LA samples (Figure 4). Therefore, the results shown in Figure 3 together with the results presented in Figure 4 suggest that H_2_S is the first product of non-enzymatic light-dependent decomposition of LA, which is, probably, next oxidized to sulfane sulfur-containing compound(s). The limitations of our study result from analytical methods used in this experiment. The colorimetric and fluorimetric methods have poor power, but they still are widely used by laboratories. In order to confirm our hypothesis, using better analytical methods (i.e., high performance liquid chromatography (HPLC), mass spectrometry (MS)) is planned for the future.

H_2_S was shown for the first time to play an important role in the regulation of many physiological processes in 1996 [33]. It is synthesized in vivo from l-cysteine and/or l-homocysteine by cystathionine β-synthase (EC 4.2.1.22; CBS), cystathionine γ-lyase (EC 4.4.1.1; CSE) and cysteine aminotransferase (EC 2.6.1.3; CAT) in cooperation with 3-mercaptopyruvate sulfurtransferase (EC 2.8.1.2; MST). H_2_S can be stored in cells in the form of sulfane sulfur. On the other hand, it can be released from sulfane sulfur-compounds [34,35,36]. Sulfane sulfur is a labile reactive sulfur atom in the 0 or –1 oxidation state and does not exist in the free form but is always attached to another sulfur atom. The outer sulfur atom of thiosulfate (S=SO_3_^2−^) and elemental sulfur (S_8_) possess these properties. Among other sulfane-sulfur containing compounds, the following should be mentioned: persulfides (R–S–S–H, HSSH), organic (R–S_n_–S–R) and inorganic polysulfides (H_2_S_n_), polythionates (^−^SO_3_–S_n_–SO_3_^−^) and disulfides in which the C–S bond is adjacent to an unsaturated carbon, such as disulfides of mercaptopyruvate, allyl mercaptan and β-mercaptoketimine [34].

The balneological procedures with the use of sulfide/hydrogen sulfide mineral waters are commonly used in the treatment of a variety of dermatological diseases, including psoriasis, atopic dermatitis, dermatoses with congenital hyperkeratosis and even scleroderma [37]. On the other hand, recent observations documented a beneficial effect of many formulations containing LA on the skin [38]. The comparison of the effects of LA and H_2_S/sulfane sulfur on the skin showed striking analogies. Namely, Tsou et al. demonstrated that LA could prove to be an effective treatment in patients with systemic sclerosis (SSc) that is a connective tissue disease characterized by fibrosis of the skin and organs [39]. On the other hand, Wang et al. reported the beneficial effects of H_2_S on SSc-associated skin and lung fibrosis [40].

Thus, the hypothesis that beneficial effects observed after treatment with various types of LA-containing cosmetic and dermatological formulations applied topically on the skin result from a direct action of H_2_S/sulfane sulfur released in the presence of environmental light from LA contained in these formulations (cream, gel, liniment, balm, lotion, ointment, etc.) seems convincing and certainly deserves further attention. More studies in this direction are needed that will significantly contribute to a better understanding of LA and the mechanisms of its pharmacological activity, thus supporting progress in the treatment of dermatological diseases.

Is LA a molecule which, like thiocysteine, can release H_2_S/sulfane sulfur in tissues? The answer to this question seems negative most of all because, in contrast to thiocysteine, LA does not possess sulfane sulfur in its structure. On the other hand, this process would be possible, provided that human body contains an enzyme(s) that extracts sulfane sulfur from thiol compounds. It is known that this kind of enzymes extract sulfane sulfur from cysteine forming alanine and are present in plant and bacterial cells [34]. It should be noted that it has never been studied whether LA is a source of the H_2_S and sulfane sulfur in animal tissues. It is known that the LA/DHLA system plays a role in H_2_S production with participation of sulfurtransferases, such as MST and thiosulfate:cyanide sulfurtransferase (EC 2.8.1.1; rhodanese, TST). Nagahara et al. demonstrated that cytosolic MST was evolutionarily related to mitochondrial TST [41]. TST- and MST-catalyzed reactions of sulfane sulfur transfer yield an unstable sulfane sulfur-containing persulfide with the –SH group of Cys-247 located in the active center of these enzymes (TST(MST)-Cys_247_–S–SH). Next, DHLA accepts a sulfur atom from TST(MST)-Cys_247_–S–SH what results in formation of DHLA hydropersulfide, from which sulfane sulfur is then released in the form of H_2_S, and LA is produced concomitantly. The first papers, in which authors demonstrated that under in vitro conditions (without tissue homogenate) DHLA served as a sulfane sulfur acceptor in TST-catalyzed reactions, were published in the 1960s [42,43]. The next paper addressing this issue was not published until 2011, when authors using the mouse brain homogenate demonstrated that, like for TST, DHLA was also a substrate for MST [44].

Our results indicated that, in the presence of rat liver (Figure 5A) and kidney homogenate (Figure 5B), the level of H_2_S in control samples was lower than in samples containing the same amounts of homogenate and LA/DHLA. However, in the case of sulfane sulfur, the relationship was opposite—namely, its level in control homogenates was higher than in the same samples containing LA/DHLA (Figure 5C,D). Thus, the H_2_S production is associated with a decrease of endogenous sulfane sulfur under the influence of DHLA.

**Figure 6:**
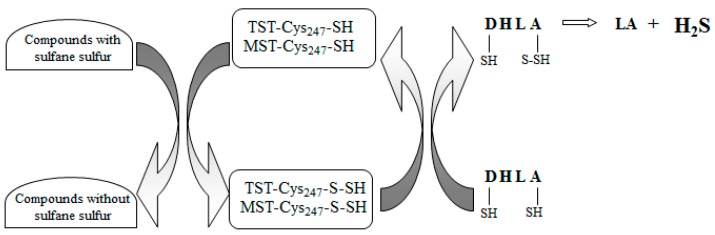
Role of LA/DHLA system in the interconversion of H_2_S and sulfane sulfur in the rat liver and kidneys. DHLA accepts sulfur atoms from sulfane sulfur-containing persulfide group of Cys-247, located in the active center of 3-mercaptopyruvate sulfurtransferase (MST) and/or thiosulfate:cyanide sulfurtransferase, rhodanese (TST). In this process, DHLA hydropersulfide is formed from which sulfur is released in the form of H_2_S, and LA is produced concomitantly (according to [44], modified).

Based on the above-discussed literature data, it seems that H_2_S is released from sulfane sulfur in tissues (liver and kidney) with participation of MST and/or TST in the presence of DHLA. Simultaneously, it means that LA is not a molecule which, like, e.g., the thiocysteine, can release H_2_S/sulfane sulfur in tissues. A putative mechanism of H_2_S formation from sulfane sulfur in the liver and kidneys in the presence of DHLA is shown in Figure 6.

## 5. Conclusions

The results indicate that both H_2_S and sulfane sulfur are formed from LA non-enzymatically in the presence of environmental light. These results suggest that H_2_S is the first product of non-enzymatic light-dependent decomposition of LA that is, probably, next oxidized to sulfane sulfur-containing compound(s).

The study in the presence of tissue homogenates indicated that LA and DHLA are not molecules which can directly release H_2_S/sulfane sulfur in tissues. It seems that, in these conditions, DHLA acts as a reducing agent that releases H_2_S from sulfane sulfur-compounds.

The same mechanism may occur in in vivo processes, which is important for a better understanding of the mechanisms of pharmacological action of LA.
